# Enhancing Type 2 Diabetes Care With CGM Integration: Insights From an Italian Expert Group

**DOI:** 10.1002/dmrr.70059

**Published:** 2025-06-11

**Authors:** Concetta Irace, Angelo Avogaro, Federico Bertuzzi, Raffaella Buzzetti, Riccardo Candido, Stefano Del Prato, Paolo Di Bartolo, Paolo Fiorina, Carlo Bruno Giorda, Francesco Giorgino

**Affiliations:** ^1^ Department of Health Science University Magna Græcia Catanzaro Italy; ^2^ Department of Medicine University of Padua Padua Italy; ^3^ Division of Diabetology ASST Grande Ospedale Metropolitano Niguarda Milan Italy; ^4^ Department of Experimental Medicine Sapienza University of Rome Rome Italy; ^5^ Department of Medical Surgical and Health Sciences University of Trieste SC Patologie Diabetiche Azienda Sanitaria Universitaria Giuliano Isontina Trieste Italy; ^6^ Centro di Ricerca Interdisciplinare “Health Science” Scuola Superiore Sant’Anna Pisa Italy; ^7^ Department of Specialist Medicines Ravenna Diabetes Center Romagna Local Health Authority Ravenna Italy; ^8^ Division of Endocrinology ASST Fatebenefratelli‐Sacco Milan Italy; ^9^ Department of Biomedical and Clinical Science L. Sacco International Center for T1D Pediatric Clinical Research Center “Romeo ed Enrica Invernizzi” University of Milan Milan Italy; ^10^ Nephrology Division Boston Children's Hospital Harvard Medical School Boston Massachusetts USA; ^11^ Diabetes and Endocrine Disorders Turin Italy; ^12^ Department of Precision and Regenerative Medicine and Ionian Area Section of Internal Medicine, Endocrinology, Andrology and Metabolic Diseases University of Bari Aldo Moro Bari Italy

**Keywords:** basal insulin, clinical scenario, continuous glucose monitoring, cost‐effectiveness, drug therapy, multiple daily insulin injections, type 2 diabetes

## Abstract

Type 2 diabetes (T2D) is a pandemic and strongly impact patients' prognosis. Several barriers may hamper the achievement of good glycaemic control, which is the aim of diabetes care. These include but are not limited to poor treatment adherence, poor self‐management, and heterogeneity of the disease context. Diabetes self‐management is critical, particularly in insulin‐treated patients and it is largely based on glucose monitoring, which allows recording glucose levels to make informed decisions with respect to meals, exercise, and other daily‐life activities. For decades, glucose monitoring has been based on self‐measurement of capillary blood glucose, which has some obvious important limitations. With the start of the new century, systems for continuous glucose monitoring (CGM) have become available. These systems measure subcutaneous interstitial glucose levels in a continuous or intermittent manner. They allow a better description of daily glucose pattern and glycaemic trend, a more accurate identification of glucose peaks and identification of otherwise unrecognised hypoglycaemic episodes, and a more reliable assessment of the stability of glycaemic control. CGM has been repeatedly shown to improve glycaemic control and reduce the risk of hypoglycaemia in type 1 diabetes (T1D). Over the years however, evidence has been gathered on the CGM use in T2D on different treatment regimens and wider applications are clearly desired. The aim of this expert opinion paper is to summarise the currently available evidence on CGM use across the whole spectrum of T2D and suggest practical indications beyond current guidelines.

## 1 Introduction

Type 2 diabetes (T2D) affects a significant proportion of the global population and markedly elevates the risk of cardiovascular events, microvascular complications, premature mortality, and disability [1, 2, 3]. In 2020 3.5 million Italian adults had diagnosed T2D, that is, a 5.9% prevalence of the adult population, but it is higher in the elderly (21% above 75 years of age) and within the socio‐economically disadvantaged population [4].

Over the past two decades, advancements in pharmacologic therapies have offered the opportunity to enhance the clinical management of T2D. Innovative treatments, such as incretins and SGLT2 inhibitors, have been shown to be efficacious and safe with meaningful improvement of glycaemic control, low risk of hypoglycaemia, and body weight reduction. Moreover, in dedicated cardiovascular outcomes trials, these agents have been shown to confer unprecedented cardiorenal protection [5, 6]. Despite improved pharmacologic intervention, no more than 50% of the adult US diabetic population reaches an HbA1c < 7% and 25% has HbA1c > 8% [1]. Italian epidemiologic data about T2D suggest that in 2022 the mean HbA1c was 7.2%, ranging from 6.2% in patients on dietary treatment up to 7.9% in those on insulin therapy [7]. More in detail, 45% of patients have HbA1c > 7.0% and 18% have HbA1c > 8.0%, of whom 7% have HbA1c > 9.0% [7].

Concurrently, healthcare expenditures for T2D remain exceedingly high [8]. Based on a probabilistic study taking into account direct (drugs, hospitalizations, monitoring and adverse events) and indirect (absenteeism and early retirement) costs, the total economic burden of diabetes in Italy amounted to €20.3 billion/year, 54% of which was accounted for by indirect costs [9].

## 2 Barriers in the Control of Type 2 Diabetes

Several barriers may hamper the achievement of good glycaemic control. These include, but are not limited to, poor treatment adherence, poor self‐management, and heterogeneity of the disease context.

A retrospective analysis of the MarketScan Research database examining > 100,000 adults with T2D revealed that in the first year of treatment, only 65.4% had an adherence ≥ 80%, with 6.7% of the study population with < 20% adherence. The results were worse in younger people and in those with comorbidities [10]. Poor adherence has been estimated to account for about 50% of treatment failure and 75% of the gap in glycaemic control existing between randomized trials and real‐world data [11, 12]. As a consequence, poor adherence confers a 2.5‐fold higher risk of hospitalisation [13] and not achieving glycaemic targets increases the likelihood of long‐term complications, more hospitalizations, higher health care costs, and elevated mortality rates [14, 15, 16, 17].

The determinants of medication nonadherence pertain to people with diabetes, providers, as well as external factors. Patient‐related factors can be further sub‐divided into demographic (age, sex, education, employment status, income level, family size, marital status), sociocultural (health literacy, medication beliefs, perceived threat, disease perception, social network), and behavioural factors (cognitive function, mental illness, stress, substance abuse). As far as providers are concerned, poor communication and relationship with the person with diabetes should be considered. External factors include features of the disease (quiescence/severity, duration, treatment response), properties of the medications (efficacy, adverse event, regimen complexity, storage requirements), as well as system components (access to care, cost/copay, access to technology, health insurance, transitions of care) [18]. To improve treatment adherence of the people with diabetes, some strategies have been developed to overcome these barriers. These strategies include counselling, engagement of familiars and caregivers, improved patient‐diabetologist communication as well as automated alerts, tele‐ or self‐monitoring, and therapeutic regimen revision [18].

Diabetes self‐management is critical, particularly in insulin‐treated patients and it is largely based on glucose monitoring, which allows recording glucose levels to make informed decisions with respect to meals, exercise, and other daily‐life activities. For decades, glucose monitoring has been based on self‐measurement of capillary blood glucose (SMBG). Though potentially informative, the traditional procedure represents a barrier to the achievement of glycaemic control. Indeed, it is not well accepted by people with diabetes because it requires finger pricking, which can be painful, frustrating, and cumbersome [19, 20]. In addition, SMBG provides limited information on daily glucose profiles and even less with respect to various components of glucose control [21]. An Italian observational study carried out in 21 Italian centres encompassing 13,331 people with T2D and > 8.44 million SMBG values showed that patients poorly tested fasting blood glucose (FBG) and even more postprandial blood glucose (PPG) irrespective of their therapeutic regimen (including insulin therapy) [22].

**FIGURE 1:**
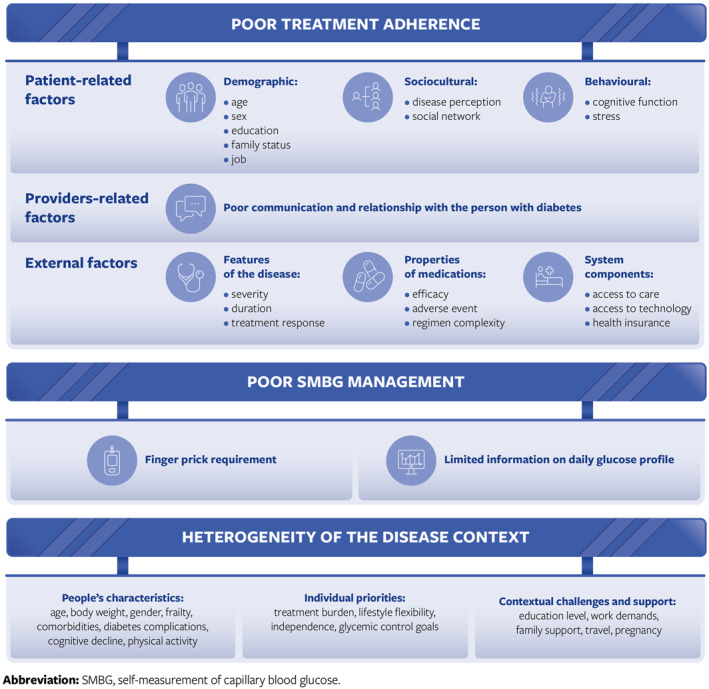
Barriers in the control of type 2 diabetes.

Another barrier to effective diabetes control is the heterogeneity of the disease context. T2D is a complex condition, often requiring multifactorial behavioural and pharmacological treatments in an attempt to achieve a glycaemic target, avoid complications, and improve quality of life (QoL) [23]. People's characteristics and their desires and expectations vary widely. Age, education, occupation, family environment, travelling, pregnancy and much more contribute to such a heterogeneity (Figure 1).

With the start of the new century, systems for continuous glucose monitoring (CGM) have become available. These systems generally measure subcutaneous interstitial glucose levels in a continuous or intermittent manner. They allow a better description of daily glucose pattern and glycaemic trend, a more accurate identification of glucose peaks and identification of otherwise unrecognised hypoglycaemic episodes, and a more reliable assessment of the stability of glycaemic control. Their use has resulted in new glucose metrics such as time in range (TIR) or below (TBR)/above range (TAR), glucose variability, and number of episodes of hypo‐ and hyperglycaemias. All these parameters were evaluated in a specific time range of 2 or 4 weeks [24]. Initially adopted for people with type 1 diabetes (T1D), CGM has been repeatedly shown to improve glycaemic control and reduce the risk of hypoglycaemia. Over the years, however, evidence has been gathered on the CGM use in T2D on different treatment regimens [25].

Aim of this paper is to summarise the currently available evidence on CGM use across the whole spectrum of T2D and suggest practical indications beyond current guidelines.

## 3 Guidelines and CGM Use in T2D

Continuous glucose monitoring is becoming a cornerstone in the management of diabetes. International and National guidelines suggest, in addition to T1D, the implementation of the CGM in T2D in specific settings.

Very recently, the American Diabetes Association (ADA) revised its clinical guidance, recommending the use of real‐time or intermittently scanned continuous glucose monitoring (CGM) in both youth and adults with diabetes receiving any form of insulin therapy. The decision regarding the most appropriate CGM device should be individualised, taking into account the patient's clinical profile, lifestyle, and preferences. The updated recommendations also support considering CGM in adults with T2D managed with glucose‐lowering therapies other than insulin as a means to help achieve and sustain personalised glycaemic targets. In all cases, device selection should reflect the unique needs and circumstances of each patient [26]. A similar recommendation for individuals with T2D comes from the American Association of Clinical Endocrinologists (AACE) [27]. The consensus report by the ADA and the European Association for the Study of Diabetes (EASD) suggests considering CGM in people with T2D who are on insulin [23].

The recent update of the NICE guidelines recommends the use of the flash glucose monitoring in individuals with T2D aged more than 18 years who use insulin two or more times each day and if they have recurrent or severe hypoglycaemia, impaired hypoglycaemia awareness, disability limiting SMBG or if more than 8 glucose tests per day are needed [28].

The last version of the Diabetes, Cardiorenal, and/or Metabolic (DCRM) Multispecialty Practice Recommendations suggests the continuous use of CGM in all people with T2D on insulin and considering its use for those on sulfonylureas in those with hypoglycaemia. Also, episodic use is supported as an audit of glycaemic patterns in any person with diabetes taking any antihyperglycaemic medication as well as in persons desiring information on the impact of diet and physical activity [29].

## 4 CGM Evidence in Type 2 Diabetes

SMBG obsolescence and the limitations of HbA1c [30] underscore the need for using new tools and glucometrics to improve glycaemic control and therapeutic management throughout the clinical history of a patient with T2D [25, 31].

The CGM systems act through sensors inserted transcutaneously or subcutaneously to measure glucose levels in the interstitial fluid. Two types of CGM systems are currently available: real‐time CGM (rtCGM) and intermittently scanned CGM (isCGM), also called Flash Glucose Monitoring (FGM). Both systems provide information about current and previous glucose levels, as well as glucose trends and anticipated future glycaemic status. Moreover, both rtCGM and isCGM sensors collect continuous real‐time glucose readings. isCGM provides this information each time the user actively scans the sensor with the device reader or via an app on a smartphone, whereas rtCGM passively transmits this information without user engagement. In addition, the isCGM reader is itself a blood glucose meter, making capillary blood glucose measurement readily available if needed [32].

CGM systems are designed to reduce the burden of glucose monitoring and provide reliable monitoring of daily glucose fluctuations and responses to insulin [33]. A recent meta‐analysis included 26 RCTs (17 on CGM and 9 on isCGM) involving 2783 patients with T2D (CGM 632 vs. usual care/SMBG 514 and isCGM 871 vs. usual care/SMBG 766); it showed an indirect comparison in terms of efficacy between the two devices: considering that the context of use is clinically different, the efficacy seems higher with isCGM. Device‐related adverse events, however, are logically similar [34].

According to a recent retrospective study carried out in a large cohort (N 30,585) of people with T2D, the prevalence of CGM use is 12.7%, mainly in young individuals on insulin treatment with a progressive increase in monthly CGM adoption from year 2020 through 2021 [35]. CGM utilization in Italy, however, remains limited. In 2018, among 438,306 individuals with T2D, only 5518 (1.2%) were on CGM [36].

So far, numerous studies have been published reinforcing the role of CGM in T2D in different settings. We have commented on some of the studies (reported in chronological order) which may support the scope of the study.

### 4.1 Type 2 Diabetes on Multiple Daily Insulin Injection (MDI)

The Replace study [37] is a multicentre, open‐label randomized controlled trial that recruited T2D adults on intensive insulin treatment with an average baseline HbA1c of 8.6%. The primary endpoint was the difference in HbA1c change after 6 months using isCGM (*n* = 149) or SMBG (*n* = 75). The protocol procedure required participants in the CGM arm to use the sensor glucose data for self‐management, including insulin dose titration although no training was provided to the participants for interpreting the glucose sensor data. In the isCGM group, the HbA1c change from baseline was −0.29% with no difference from what was obtained using SMBG (−0.31 ± 0.09%). When people under the age of 65 were analysed, a greater HbA1c drop (−0.53%) was apparent in those using the isCGM system compared to those using SMBG (−0.20 ± 0.12%; *p* = 0.0301). The number of hypoglycaemic episodes significantly decreased by 27.7% in the isCGM group irrespective of age and the average duration of hypoglycaemia went from the initial 1.3 h per day to 0.59 h per day after 6 months (average change: −0.47 h) in the intervention group and from 1.08 h per day to 0.99 h per day in the control group. The difference between the two groups was −0.47 h (SE 0.134), that is, a 43% reduction in the period of hypoglycaemia (*p* = 0.0006).

The DIAMOND study [38] is a multicentre randomized, controlled trial evaluating the effect of rtCGM versus usual care (1:1) in 158 people with longstanding T2D (median of 17 years) on MDI treatment regimen. The primary outcome was HbA1c reduction at 24 weeks. Participants received general instruction on how to use CGM under their physicians' supervision. From an average baseline of 8.5%, HbA1c levels decreased to 7.7% in the CGM group and to 8.0% in the control group (adjusted difference in mean change, −0.3% [95% CI, −0.5% to 0.0%]; *p* = 0.022) with no apparent difference with respect to hypoglycaemia or quality‐of‐life outcomes.

The study by Yaron et al. [39] is a randomized, controlled trial evaluating changes in HbA1c, rates of hypoglycaemia, and QoL in 101 people with T2D randomized to use isCGM or SMBG over a 10‐week follow‐up. Participants in the isCGM arm were instructed to scan the sensor at least every 8 h and all participants received the same diabetes management instruction for carbohydrate counting and insulin dose adjustment. At the end of the study period, HbA1c was −0.82% and −0.33% in the isCGM and SMBG groups versus baseline, respectively (*p* = 0.005). In non‐predefined post hoc analyses, 68.6% of those on isCGM had a HbA1c reduction > 0.5% compared with 30.2% in the SMBG group (*p* < 0.001), and 39.2% in the isCGM group had a reduction > 1.0% versus 18.6% in the control group (*p* = 0.0023) without increased frequency of hypoglycaemia. The isCGM group found the treatment more flexible (*p* = 0.019), were more willing to recommend it (*p* = 0.023) and reported higher treatment satisfaction.

The RELIEF study [40] carried out on the national French database assessed the impact of isCGM in 74,011 persons with T1D (45%) and T2D (55%). Among them, 88% were on MDI or CSII treatment, and 12%, almost all individuals with T2D, were on basal insulin or oral hypoglycaemic drugs. The study showed a reduction in the number of hospitalizations for acute diabetes complications in both T1D (−49.0%) and T2D patients (−39.4%) following the use of isCGM, as compared to the period prior to the initiation of the isCGM, over a 12‐month observation period. Similarly, in the isCGM group, a reduction in hospitalizations for hypo‐ and hyperglycaemia in T2D was found (−10.8% and −26.5%, respectively). Similar benefits were confirmed at the end of the second year of isCGM use [41].

Another real‐life retrospective analysis carried out in North California in 3806 people using various CGMs reported a mean HbA1c reduction of 0.40% (*p* < 0.001), along with fewer hypoglycaemic events (−2.7%, *p* = 0.001) and outpatient or telephone visits [42].

Similarly, among 10,370 isCGM users in the UK, a significant improvement in glycaemic control (−5.2 mmol/mol change in HbA1c after 7.5 (interquartile range 3.4–7.8) months of follow‐up) and hypoglycaemia awareness and a reduction in hospital admissions were demonstrated [43].

The Prefer IT study [44] is an Italian multicentre observational cohort study that measured the change in HbA1c in T2D people with and without isCGM, all receiving non‐standardised recommendations. The study included 322 patients, 109 with isCGM and 213 with SMBG. After 3–6 months, in the 234 completers (83 isCGM, 151 SMBG), HbA1c reduction in isCGM was significantly greater compared with SMBG (0.3% ± 0.12, *p* = 0.0112), a difference that remained significant after adjustment for confounding factors.

In summary, the examples summarised above and in Table 1 are in line with those obtained in people with T1D and support the use of CGM in people with T2D on MDI. Accordingly, in Italy, CGM is currently reimbursed for these individuals.

**TABLE 1:** Clinical studies of CGM use in type 2 diabetes on multiple daily insulin injection (MDI).

Reference	Patient population	CGM/FGM	Study design	Outcomes	Results
Haak et al. [37]	Type 2 diabetes on MDI (*n* = 224)	Free Style Libre	RCT: BGM versus CGM	Glycaemic control at 6 months	Time in hypoglycaemia −43% Time in hypoglycaemia at night −54%
Beck et al. [38]	Type 2 diabetes on MDI (*n* = 158)	Dexcom G4 Platinum	RCT: BGM versus CGM	Glycaemic control at 6 months	HbA1c −0.3% HbA1c reduction of ≥ 1.0% + 20%
Yaron et al. [39]	Type 2 diabetes on MDI (*n* = 101)	Free Style Libre	RCT: BGM versus CGM	Satisfaction and glycaemic control at 10 weeks	Treatment significantly more flexible with CGM Reduction HbA1c ≥ 0.5% in 68.6% (*p* < 0.001) and ≥ 1.0% in 39.2% (*p* < 0.0023)
Roussel et al. [40]	Type 2 diabetes on any therapy (*n* = 40,846); 88% on MDI	Free Style Libre	Retrospective observational	Diabetes related hospitalizations at 1 year (compared with the last year before CGM)	Hospital admissions for acute diabetes mellitus events −39.4% Hospital admissions for DKA −52.1% Hospital admissions for hypoglycaemia −10.8%
Riveline et al. [41]	Type 2 diabetes on any therapy (*n* = 40,846); 88% on MDI	Free Style Libre	Retrospective observational	Diabetes related hospitalizations at 1 year (compared with the last year before CGM)	Hospital admissions for acute diabetes mellitus events −48% Hospital admissions for DKA −47% Hospital admissions for hypoglycaemia −43%
Karter et al. [42]	Type 2 diabetes on insulin (*n* = 36,080)	Various	Retrospective cohort study: BGM versus CGM	Glycaemic control and diabetes related hospitalizations at 1 year	HbA1c −0.56% Hospital admissions for hypoglycaemia −4.0%
Deshmukh et al. [43]	Type 1 and 2 diabetes on insulin (*n* = 10,370; 97% type 1 diabetes)	Free Style Libre	Prospective	Glycaemic control and diabetes related hospitalizations at 7.5 months	HbA1c −0.5% Reduction in paramedic callouts and hospital admissions due to hypoglycaemia and hyperglycaemia/DKA
Bosi et al. [44]	Type 2 diabetes on basal insulin (*n* = 322)	Free Style Libre	Prospective observational cohort	Glycaemic control at 3–6 months	HbA1c −0.3%

Abbreviations: BGM, blood glucose monitoring; CGM, continuous glucose monitoring; DKA; diabetic ketoacidosis; MDI, multiple daily insulin injection; RCT, randomized controlled trial.

### 4.2 Type 2 Diabetes on Basal Insulin and/or Non‐Insulin Treatment

Allen et al. [45] conducted a randomized controlled trial enrolling 52 people with T2D non‐insulin treatment to evaluate changes in physical activity behaviour by using rtCGM compared with SMBG. All participants received individualised diabetes education and physical activity counselling, while participants in the intervention group received CGM for just 3 days and CGM counselling. At the end of the study (8 weeks), participants who wore CGM showed a higher level of confidence in maintaining a physical activity programme versus SMBG participants. Additionally, CGM users had a greater HbA1c reduction than non‐users (−1.16% vs. −0.32%, *p* < 0.05).

In the Guardian trial, when 65 people with poor glucose control on oral hypoglycaemic agents and/or insulin were randomized to rtCGM for intermittent monitoring (3 days a month over 3 months) or SMBG at least 4 times/week for 3 months, the former experienced a greater reduction in HbA1c after 12 weeks (−1.1% vs. −0.4%, *p* = 0.004). Interestingly, in the rtCGM group there was a significant reduction of daily calories and a significant increase in total exercise time [46].

Another randomized controlled trial including 100 people with T2D on basal insulin demonstrated that the intermittent use of rtCGM (12 weeks) determined a sustained (40 weeks) reduction of HbA1c compared to SMBG (−0.8% vs. −0.2%, *p* < 0.04) [47].

Similarly, a prospective, 52‐week, two‐arm, randomized trial comparing rtCGM (*n* = 50) used for four 2‐week cycles (2 weeks on/1 week off) versus SMBG (*n* = 50) in people with T2D not taking prandial insulin, demonstrated a significant improvement in HbA1c at 12 weeks (−1.0% ± 1.1% vs. −0.5% ± 0.8%, *p* = 0.006) [48].

More recently, 30 noninsulin—treated patients have been randomized to rtCGM and lifestyle intervention focused on the reduction of postprandial glucose excursion or SMBG and conventional medication management. After a 5‐month observation period, the rtCGM group showed a −1.11% mean reduction of HbA1c compared with SMBG (*p* = 0.03) [49].

A retrospective observational study conducted by Miller and colleagues [50] evaluated the change in HbA1c levels at 6 and 12 months after starting isCGM in people with T2D treated with basal insulin or with oral drugs ± GLP1‐RA. The analysis included 774 subjects for the 6‐month endpoint and 207 subjects for the 12‐month endpoint. At 6 months, a reduction in the HbA1c level of—0.8% was observed and at 12 months of—0.6%. The main reduction was observed in the group of patients not on insulin (−0.9% at 6 months and −0.7% at 12 months).

In a randomized clinical trial [51] carried out at 15 U.S. primary care centres 175 patients treated with basal insulin were randomized to CGM versus SMBG (2:1). All participants received general diabetes education according to the centre's usual diabetes educational programme. The primary outcome was HbA1c level at month 8 and key secondary outcomes were CGM‐measured time in target glucose range (70–180 mg/dL), time spent at glucose level >250 mg/dL, and mean glucose level. Mean HbA1c level decreased from 9.1% to 8.0% in the CGM group and from 9.0% to 8.4% in the SMBG group (adjusted difference, −0.4% [95% CI, −0.8% to −0.1%]; *p* = 0.02). In the CGM group, compared with the SMBG group, mean percentage time in the target glucose range was 59% versus 43% (adjusted difference, 15% [95% CI, 8%–23%]; *p* < 0.001), mean percentage time at glucose >250 mg/dL was 11% versus 27% (adjusted difference, −16% [95% CI, −21% to −11%]; *p* < 0.001), and mean glucose values were 179 mg/dL versus 206 mg/dL (adjusted difference, −26 mg/dL [95% CI, −41 to −12]; *p* < 0.001).

Another multicentre trial [52] explored the effect of discontinuing CGM after 8‐month use in adults with T2D on basal insulin. After the initial randomisation to rtCGM and blood glucose monitoring (BGM), participants in the CGM group were randomly reassigned to continue either CGM (*n* = 53) or to CGM discontinuation with resumption of BGM (*n* = 53). The BGM group continued monitoring glucose (*n* = 57) for the entire duration of the study. In the CGM discontinuation group, TIR increased from 38% to 62% after 8 months and decreased to 50% after the additional 6 months. In the group which continued CGM, the initial 8‐month positive effect did not further change after 6 months (44%, 56%, 57%). Interestingly, comparing the two groups at 14 months, the treatment group difference in mean TIR was not statistically significant. This result suggests the potential long‐lasting beneficial effect of CGM use, possibly due to CGM‐induced lifestyle and diet modifications, as well as improved medication adherence favoured by a better confidence with disease management.

A retrospective observational study conducted on a national US database assessed the effect of CGM on HbA1c in poorly controlled people with T2D on treatment with basal insulin [53]. The study included people with isCGM prescriptions from October 2017 through February 2020. The study cohort included 1034 subjects with an average age of 51.6 years. After 6 months, HbA1c was reduced by −0.8% and after 12 months by −0.6%. The greatest HbA1c reduction occurred in participants with HbA1c at baseline ≥ 12.0% (*n* = 181, −3.7%) and in noninsulin—treated participants either after 6 months (*n* = 497, −0.9%) or 12 months (*n* = 120, −0.7%).

The study by Choe et al. [54] is an open‐label randomized controlled trial comparing isCGM and SMBG in people with T2D treated with basal insulin or oral drug therapy. More in detail, the authors investigated the effect of patient‐driven lifestyle modification using isCGM based on a simple dietary advice to enhance consumption of healthy food with the low glycaemic index and avoid food responsible of post‐meal high glucose. After 12‐week treatment, isCGM use led to a significantly greater reduction in HbA1c (risk‐adjusted difference −0.50% [95% CI −0.74 to −0.26]; *p* < 0.001) and body weight as compared to SMBG. Furthermore, the proportion of study participants achieving HbA1c < 7.0% at 12 weeks was higher with isCGM than with SMBG (14% vs. 5%).

A real‐world study conducted on 711 T2D people aged 18 and > 75 years based on the data from the Swedish national diabetes registry showed a significant and persistent reduction in HbA1c after 6‐ and 12‐month isCGM use (−0.50% and −0.52% respectively, *p* < 0.0001). Reduction of HbA1c was observed among people who were truly naïve to isCGM or had unknown prior experience and among those with greater baseline HbA1c. Interestingly, HbA1c change occurred in a wide age range (25–74 years) [55].

Within the French RELIEF study [56], among 5933 people with T2D on basal insulin, in the year after isCGM prescription, as compared to the year before, the rate of hospitalizations for ketoacidosis was 75% lower. Similarly, hospitalizations for severe hypoglycaemia dropped by 44% and those for diabetes‐related coma by 71%, along with a 7% reduction in hospitalizations for all causes. These benefits persisted at the end of the second year as well, with a further 43% reduction in hospitalizations for ketoacidosis.

Evaluation of glycaemic control in the short and medium term after myocardial infarction by using isCGM or SMBG was the aim of the LIBERATES trial [57]. The study is a multicentre two‐arm randomized trial performed in 141 individuals with T2D with recent‐onset acute myocardial infarction treated with insulin and/or sulfonylurea before hospital admission. In these people, the use of isCGM was associated with 17 min/day increase in TIR and 80 min/day less time spent at lower (< 3.9 mmol/L) glucose levels at days 76–90 after myocardial infarction. Compared with baseline, HbA1c showed similar reductions of 7 mmol/mol at 3 months in both study arms. QoL measures marginally favoured isCGM. Finally, the intervention proved to be cost effective. No specific education was provided to individuals in the isCGM group compared with the SMBG group. All insulin‐treated patients were instructed to titrate insulin according to fasting and pre‐meal glucose levels.

Very recently, an observational, retrospective, real‐world study was performed in Italy in 132 people with T2D on basal insulin or oral antidiabetic drugs starting with isCGM [58]. Participants were mainly male (69.5%), with a mean age of 68.2 ± 11.0 years, disease duration 19.0 ± 9.4 years (79.7%), with or without concomitant non‐insulin therapy and a mean baseline HbA1c of 8.1 ± 1.3%. The estimated mean HbA1c reduction was statistically significant at 3 (−0.4 ± 1.0%; *p* = 0.003) and 6 months (−0.6 ± 1.3%; *p* < 0.0001).

A multicentre, open label, randomized, parallel group study compared the effect of 24‐week isCGM versus SMBG on the glycaemic control of people with T2D not using insulin [59]. Participants in both arms were educated on the use of the devices and about diet and lifestyle adjustments. HbA1c decreased by −0.46% (−5 mmol/mol, *p* < 0.001) and −0.17% (−1.8 mmol/mol, *p* = 0.124) respectively in the isCGM and SMBG groups. In line with HbA1c, individuals in the isCGM group showed a significantly lower percentage of TAR, glycaemic variability and higher percentage of TIR. Furthermore, the use of isCGM was associated with increased treatment satisfaction and it was perceived as convenient and painless.

Data on T2D subjects not on insulin treatment were collected in a retrospective observational study conducted to evaluate the clinical consequences of isCGM [60]. In a cohort of 10,282 subjects with an average age of 53.1 years (±9.6), over a 6 month follow‐up period, the rate of adverse diabetes‐related events and all‐cause hospitalisation decreased from 0.076 to 0.052 events/patient/year (HR 0.68; 95% CIs 0.58–0.80; *p* < 0.001) and from 0.177 to 0.151 events/patient/year (HR 0.85; 95% CIs 0.77–0.94; *p* = 0.02), respectively. These results suggest that the use of isCGM in noninsulin—treated T2D individuals improves clinical acute outcomes and reduces the costs associated with diabetes for the health care system.

A recent study from Canada [61], IMMEDIATE study, randomized 116 people with noninsulin—treated T2D to isCGM and diabetes self‐management education (DSME) versus DMSE alone. All participants were instructed to self‐monitor their blood glucose at least 4 times daily, supported by both scheduled learning exercises and unscheduled reminders focussing on glucose self‐monitoring. The main finding of the study was the significant improvement of TIR and TAR in the isCGM + DMSE group compared with DMSE after 16‐week intervention. The effect on TIR was independent of number or hypoglycaemic drugs and GLP‐1 RA treatment. In this study, for the first time was evaluated the time spent in the tight glucose range (TITR, 70–140 mg/dL, 3.9–7.8 mmol/L). The adjusted mean difference between groups was −8.5% (95% CI, −16.6% to −0.03%; *p* = 0.042). The trial also showed that isCGM + DMSE was associated with a greater HbA1c reduction from baseline than education alone (adjusted mean difference −0.3% (3 mmol/mol) (95% CI, 0%–0.7%; *p* = 0.048)).

More recently, a large (*N* = 24,246 individuals) real‐world study has assessed the impact of isCGM in T2D adults who are suboptimally controlled and treated with a GLP‐1 RA formulation. After 6 months, the combination of GLP‐1 RA + isCGM significantly improved HbA1c compared with a matched cohort of GLP‐1 RA alone users (GLP‐ 1 RA, *N* = 478, difference −2.43%; GLP‐1 RA alone, *N* = 2390, difference −2.06%). The effectiveness of the combination of GLP‐1 RA + isCGM was confirmed in the sub‐analysis including individuals who were on intensive or non‐intensive additional insulin treatment [62].

Overall, these studies provide the evidence (resumed in Table 2) that both rtCGM and isCGM can be instrumental in improving glycaemic control in people with T2D on basal insulin and/or hypoglycaemic drugs. Some studies have also specifically evaluated the effect of CGM use on changes in lifestyle and dietary habits.

**TABLE 2:** Clinical studies of CGM use in type 2 diabetes on basal insulin and/or non‐insulin treatment.

Reference	Patient population	CGM/FGM	Study design	Outcomes	Results
Allen et al. [45]	Type 2 diabetes on non‐insulin (*n* = 52)	Not specified	RCT: BGM versus CGM	Glycaemic control and BMI control at 8 weeks	HbA1c −1.16% versus −0.32% BMI −0.53 kg/m^2^ versus −0.12 kg/m^2^
Yoo et al. [46]	Type 2 diabetes on any therapy (*n* = 65); 56% on insulin	Guardian RT	RCT: BGM versus CGM	Glycaemic control and life style at 12 weeks	HbA1c −1.1% versus −0.4% Reduction in total daily calorie intake Body weight Increased exercise time
Vigersky et al. [47]	Type 2 diabetes on basal insulin (*n* = 100)	Dexcom SEVEN	RCT: BGM versus CGM	Glycaemic control at 1 year	HbA1c −0.8% versus −0.2%
Ehrhardt et al. [48]	Type 2 diabetes on oral drugs or basal insulin (*n* = 100)	Dexcom SEVEN	RCT: BGM versus CGM	Glycaemic control at 12 weeks	HbA1c −0.5% HbA1c reduction greater in those using CGM for ≥ 48 days
Cox et al. [49]	Type 2 diabetes on non‐insulin (*n* = 30)	Dexcom G4 or G5	RCT: BGM versus CGM	Glycaemic control at 5 months	HbA1c −1.11%
Miller et al. [50]	Type 2 diabetes on oral drugs or basal insulin (*n* = 774)	Free Style Libre	Observational retrospective	Glycaemic control at 6 and 12 months	Hba1c −0.8% at 6 months HbA1c‐0.6% at 12 months Greater HbA1c reduction in non‐insulin group
Martens et al. [51]	Type 2 diabetes on basal insulin (*n* = 176)	Dexcom G6	RCT: BGM versus CGM	Glycaemic control at 8 months	HbA1c −0.8% TIR +27%
Aleppo et al. [52]	Type 2 diabetes on basal insulin (*n* = 163)	Dexcom G6 Pro	RCT: BGM versus CGM for 8 months, then CGM group randomized to BGM versus CGM for 6 months	Glycaemic control at 8 and 14 months	CGM discontinued: TIR +24% at 8 months, then −12% at 14 months CGM continued: TIR +12% at 8 months, then +1% at 14 months Adjusted treatment group difference in mean TIR at 14 months −6%
Wright et al. [53]	Type 2 diabetes on oral drugs or basal insulin (*n* = 1034)	Free Style Libre	Observational retrospective	Glycaemic control at 6 months	Full cohort: Hba1c −1.5% Basal insulin: HbA1c −1.1% Oral drugs: HbA1c −1.6%
Choe et al. [54]	Type 2 diabetes on oral drugs or basal insulin (*n* = 126)	Free Style Libre	RCT: Standard care + BGM versus structured education + CGM	Glycaemic control and life style at 12 weeks	HbA1c −0.50% Body weight −1.5 kg
Eeg‐Olofsson et al. [55]	Type 2 diabetes on any therapy (*n* = 711)	Free Style Libre	RCT: BGM versus CGM	Glycaemic control at 6 and 12 months (compared to 6 months before)	HbA1c −0.50% at 6 months HbA1c −0.52% at 12 months
Guerci et al. [56]	Type 2 diabetes on basal insulin (*n* = 5933)	Free Style Libre	Retrospective observational	Diabetes related hospitalizations at 2 years (compared with the last year before CGM)	Hospital admissions for acute diabetes mellitus events −63% Hospital admissions for DKA −68% Hospital admissions for hypoglycaemia −58%
Ajjan et al. [57]	Type 2 diabetes and AMI on insulin or sulfonylurea (*n* = 141)	Free Style Libre	RCT: BGM versus CGM	Glycaemic control at 90 days	Increased TIR Reduced hypoglycaemia
Conti et al. [58]	Type 2 diabetes on basal or non‐insulin therapy (*n* = 132)	Free Style Libre 2	Observational retrospective	Glycaemic control at 3 and 6 months	HbA1c −0.4% at 3 months HbA1c −0.6% at 6 months
Wada et al. [59]	Type 2 diabetes on oral drugs (*n* = 93)	Free Style Libre	RCT: BGM versus CGM	Glycaemic control at 12 weeks	HbA1c −0.29% Reduced metrics of glycaemic variability
Miller et al. [60]	Type 2 diabetes on basal or non‐insulin therapy (*n* = 10,282)	Free Style Libre 2	Observational retrospective	ADE and ACH compared 6 months prior to and 6 months post CGM acquisition	ADE −32% ACH −15%
Aronson et al. [61]	Type 2 diabetes on non‐insulin (*n* = 116)	Free Style Libre	RCT: BGM versus CGM	Glycaemic control at 16 weeks	HbA1c −0.3% (baseline adjusted) TIR +9.9% (2.4 h)
Wright et al. [62]	Type 2 diabetes on any therapy, starting GLP1ra (*n* = 24,724)	Free Style Libre	Observational prospective	Glycaemic control at 6 months	HbA1c −0.70% (unmatched cohort) HbA1c −0.37% (matched cohort)

Abbreviations: ACH, all‐cause inpatient hospitalizations; ADE, acute diabetes‐related events; AMI, acute myocardial infarction; BGM, blood glucose monitoring; BMI, body mass index; CGM, continuous glucose monitoring; GLP1ra, GLP1 receptor agonist; RCT, randomized controlled trial; TIR, time in range.

### 4.3 CGM and Type 2 Diabetes: Meta‐Analyses

Several meta‐analyses are available to explore in a more comprehensive manner the potential contribution of CGM in improving outcomes in people with T2D. A meta‐analysis on 25 studies and 1723 participants focussing on observed change in HbA1c at either 2, 3 or 4 months, in adult or paediatric T1D and T2D subjects, as well as a longitudinal analysis up to 12 months in adults, confirms that starting isCGM as part of diabetes care results in a significant and sustained reduction in HbA1c for adults and children with T1D and for adults with T2D (mean change −0.56%; 95% CI ‐0.76, −0.36). A longitudinal analysis in adult subjects (*n* = 1276) shows that HbA1c decreased within the first 2 months and changes were sustained up to 12 months [63].

The meta‐analysis by Carlson et al. [64] assessed the impact of isCGM in T2D adult subjects on basal insulin and reported an overall HbA1c reduction of 1.1% (*p* < 0.0001): from a baseline of 9.2% ± 1.0%–8.1% ± 1.1%.

A meta‐analysis of 11 trials including 1425 T2D people using rtCGM or isCGM treated with insulin and/or oral hypoglycaemic agents found that CGM significantly decreased HbA1c (mean difference −0.31 mmol/mol), time above range (−9.06%), time below range (−0.30%) and significantly increased time in range (+8.49%) compared with SMBG, particularly in insulin‐treated individuals. In the network meta‐analysis comparing different CGM devices, rtCGM was found to exert a greater effect [65].

A further meta‐analysis of 12 trials has included a total of 1248 T2D patients with different treatment strategies using rtCGM or isCGM. The authors found that CGM compared to SMBG led to an HbA1c change versus baseline of −3.43 mmol/mol (−0.31%; 95% CI: −4.75, −2.11, *p* < 0.00001). This effect was comparable in studies including individuals on insulin treatment with or without oral agents (MD −3.27 mmol/mol [−0.30%]; 95% CI: −6.22, −0.31, *p* = 0.03), and individuals using oral agents only (MD −3.22 mmol/mol [−0.29%]; 95% CI: −5.39, −1.05, *p* = 0.004). In this analysis too, rtCGM showed a trend towards a larger effect (MD −3.95 mmol/mol [−0.36%]; 95% CI: −5.46 to −2.44, *p* < 0.00001) than isCGM (MD −1.79 mmol/mol [−0.16%]; 95% CI: −5.28, 1.69, *p* = 0.31). CGM was also associated with an increase in time in range (+6.36%, *p* = 0.001) and a decrease in time below range (−0.66%, *p* = 0.02), time above range (−5.86%, *p* = 0.02) and glycaemic variability (−1.47%, *p* = 0.05). However, outcome data on incident severe hypoglycaemia are still scarce [66].

Finally, the above cited meta‐analysis of 26 trials by Seidu et al. [34] included a total of 2783 T2D patients with different treatment strategies using rtCGM or isCGM. rtCGM reduced HbA1c (mean difference −0.19% [95% CI ‐0.34, −0.04]) and glycaemic medication effect score (−0.67 [‐1.20 to −0.13]), reduced user satisfaction (−0.54 [‐0.98, −0.11]) and increased the risk of adverse events (relative risk [RR] 1.22 [95% CI 1.01, 1.47]). isCGM reduced HbA1c by −0.31% (−0.46, −0.17), increased user satisfaction (0.44 [0.29, 0.59]), improved CGM metrics, and increased the risk of adverse events (RR 1.30 [0.05, 1.62]). Neither rtCGM nor isCGM had a significant impact on body composition, blood pressure, or lipid levels.

In summary, individual randomized studies, real world data, and meta‐analyses of currently available studies (resumed in Table 3) all concur to support the use of CGM in people with T2D not on insulin to improve their glucose treatment management and increase the chances to ensure better glycaemic control.

**TABLE 3:** Meta‐analyses of CGM use in type 2 diabetes.

Reference	Patient population	CGM/FGM	Study design	Outcomes	Results
Evans et al. [63]	Type 1 or 2 diabetes on any therapy (*n* = 1470 at 2–4 months; *n* = 1276 at 12 months)	Free Style Libre	Meta‐analysis of 29 observational studies	Glycaemic control at 2–4 and 12 months	HbA1c −0.55% at 2–4 months HbA1c reduction maintained at 12 months
Carlson et al. [64]	Type 2 diabetes on basal insulin (*n* = 191)	Free Style Libre	Meta‐analysis of 2 retrospective cohorts	Glycaemic control at 90–194 days	HbA1c −1.1%
Lu et al. [65]	Type 2 diabetes on any therapy (*n* = 1425)	Various	Meta‐analysis of 11 RCTs: BGM versus CGM	Glycaemic control at 10–48 weeks	HbA1c −0.31% TAR −9.06% TBR −0.30% TIR +8.49%
Jancev et al. [66]	Type 2 diabetes on any therapy (*n* = 1248)	Various	Meta‐analysis of 12 RCTs: BGM versus CGM	Glycaemic control at 10–48 weeks	HbA1c −0.31% TAR −5.86% TBR −0.66% TIR +6.36% Glycaemic variability −1.47%
Seidu et al. [34]	Type 2 diabetes on any therapy (*n* = 2783)	Various	Meta‐analysis of 26 RCTs: BGM versus CGM	Glycaemic control at 8–35 weeks	rtCGM: HbA1c −0.19% Glycaemic medication effect score −0.67 User satisfaction −0.54 Adverse events RR 1.22 isCGM: HbA1c −0.31% User satisfaction +0.44 Glucose metrics improved Adverse events RR 1.30

Abbreviations: BGM, blood glucose monitoring; CGM, continuous glucose monitoring; RCT, randomized controlled trial; RR, relative risk; TAR, time above range; TBR, time below range; TIR, time in range.

## 5 CGM Availabilities in Italy

Prescription and reimbursement of different CGM systems in Italy is heterogeneous depending on local administrative regulations. Eligibility criteria and economic reasons account for such variability. In Italy, all Italian Regions reimburse CGM for patients with T1D and T2D on MDI, while only 6/20 (30%), namely Sicily, Lombardy, Campania, Lazio, Marche and Basilicata, reimburse it also for patients with T2D on basal bolus or non‐insulin therapies. The availability and reimbursement of CGM systems are continuously evolving across different Italian regions, reflecting changes in healthcare priorities and economic considerations along with literature evidence.

## 6 CGM in Type 2 Diabetes: Experts Perspectives

### 6.1 Key Insights for Clinical Practice

**FIGURE 2:**
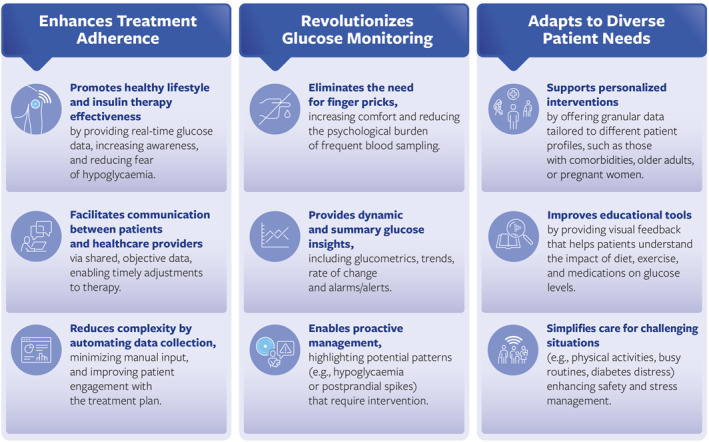
How CGM can overcome the barriers to glycaemic control.

As summarised above, a large amount of evidence suggests that CGM is beneficial for reducing the HbA1c and improve the TIR while avoiding hypo‐ and hyper‐glycaemias [67]. The benefits of CGM in T2D are not influenced by the type of treatment—whether insulin, non‐insulin, or a combination of both [61]. Available evidence recommends the use of CGM in all insulin‐treated patients (both MDI and basal) [23, 26, 27], however, a panel of experts based on its clinical experience and available data recommends intermittent use in selected cases and/or particular types of patients. Figure 2 summarises how CGM can overcome the barriers to glycaemic control by enhancing treatment adherence, revolutionising glucose monitoring, and adapting to diverse patient needs, providing a comprehensive solution to improve diabetes management.

Although the beneficial effects of CGM are quite apparent in people with T2D, intermittent use of CGM can help the person with T2D not on insulin as well [68]. In these individuals, CGM can be used for a relatively short period of time as a diagnostic tool during treatment escalation, de‐intensification, or modification to facilitate earlier reach of the target. In a narrative review of 11 studies and 5542 people with T2D, intermittent use of CGM rather than SMBG reduced HbA1c, body weight, caloric intake, and favoured adherence to dietary plan, food choice, and physical activity [69, 70]. Similarly, intermittent use of CGM in poorly controlled T2D patients improved diet and exercise habits, reducing body mass index, PPG, and HbA1c after 3‐month use [46]. Two other trials in T2D patients failing multiple non‐insulin therapies confirmed that limited episodic rtCGM use was an effective method for glucose control [71, 72]. Much of the beneficial effect of CGM is likely to be mediated by patients' empowerment allowing them to be more proactive in their daily life, helping them avoiding or correcting situations that may lead to hypo‐ and hyperglycaemia. CGM data can, indeed, be more intuitive and educational for the persons with diabetes and their caregivers. As such, the episodic use of CGM might be beneficial at the time of diabetes diagnosis to improve glycaemic stability, support patient's education and self‐knowledge, or to face the fear of hypoglycaemia [73, 74, 75].

CGM can offer a unique treatment guidance in diabetic people with complications such as a more appropriate insulin dosing in those with diabetic gastroparesis [76, 77] or in avoiding unrecognised hypoglycaemia in people on dialysis [78]. Also, in diabetic women seeking pregnancy, the short‐term CGM use may be beneficial to adapt the therapeutic regimen and achieve ideal glycaemic control [79, 80].

Intermittent CGM usage can also be of help at the time of surgery or acute events to control stress hyperglycaemia [81, 82, 83, 84, 85, 86]. Therefore, their use could be exploited under critical circumstances such as hospitalisation or to support individuals with limited self‐sufficiency.

A comprehensive algorithm about the possible intermittent use of CGM in different scenarios has been provided by Klupa et al. [67] suggesting for people with T2D a use also as ‘diagnostic biopsy’ for subjects not achieving goals despite confirmed treatment adherence. In addition, very recently a panel of European experts elaborated a consensus paper about CGM in routine care of T2D, underlining the consolidated role of rtCGM continuously used in MDI patients or those treated with basal insulin, but also the usefulness of an intermittent use of isCGM in non‐insulin‐treated ones [25].

An important feature of the CGM is the interconnectivity. Electronic data enable connectivity facilitating telemonitoring and televisits, improving rational use of time and healthcare financial resources, favouring a higher number of medical contacts and adherence, and allowing data sharing among the diabetes and outside the diabetes team. Taking advantage of this peculiarity, the Tuscany region among the first in Europe, has manage to ensure the automatic upload of the blood glucose monitoring with the FreeStyle Libre on the individual electronic chart of the person with diabetes allowing immediate access to the data to the health care providers [87]. In line with this new opportunity is observation that even professional CGM, to which the patient is blinded, can improve glucose control and serve as an educational tool [88, 89].

**FIGURE 3:**
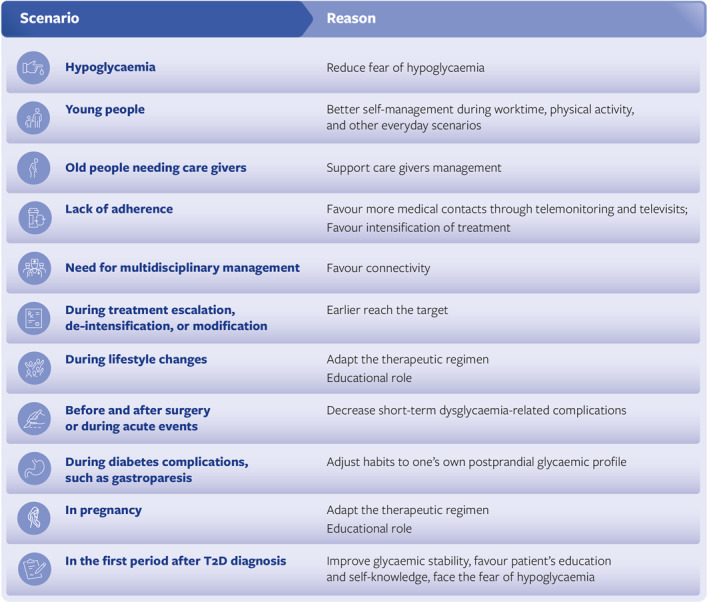
Expert suggestion on intermittent CGM use for T2D patients on oral antidiabetic drugs.

Figure 3 summarises those expert opinions, with the suggestion of a practical approach for the different clinical scenarios.

Limitations or drawbacks can occur in T2D when using CGM, possibly limiting in the long‐term the benefit the system may offer. Currently, there is no evidence about the prolonged use of CGM in T2D in terms of adherence and effectiveness. Strategies for enhancing patient adherence to maximise the long‐term benefits of this technology can be set up. To enhance adherence, it is essential to implement personalised strategies tailored to each patient's needs. First, healthcare providers should identify individual targets and discuss realistic expectations with patients, avoiding the creation of false hopes about the device's capabilities. Reducing alarm burden by limiting notifications to those that are truly necessary can help alleviate stress and prevent desensitisation to alerts. Proper education on the correct use of the device is also crucial: patients should be informed about when to scan with isCGM devices or check values with rtCGM systems, and what actions to take based on the readings. Furthermore, it is important to teach patients how to interpret data effectively, considering not only the absolute value but also the trend graph and the rate of change indicators displayed on both systems. This approach is particularly helpful for managing hypoglycaemic events. Additionally, contextualising blood glucose levels in relation to daily activities such as meals or exercise is essential; patients should be encouraged to annotate their activities for future review with healthcare providers. To further engage patients, gamification strategies can be implemented, adding an element of motivation to the process. Recognising and celebrating progress and good results will reinforce the positive behaviours necessary for sustained CGM use. By combining these strategies, healthcare providers can significantly improve CGM adherence, leading to better overall management of diabetes.

Technological limitations or even the improper or incorrect use of the device can be overcome by adequate patient or care giver education and engagement. Education includes the interpretation of glucose change in response to several routine situations such as meals, exercise, medications, stress, illness, menses, sedentariness, and leisure. Equally important is the education on the correct use of the device as the number and the timing of scan or visualisation per day, the interpretation of the trend arrow, glucose pattern, and glucose variability interpretation, the periodic evaluation of the new metric (TIR, TAR, TBR), the analysis of historical data in the short and long‐term, and the appropriate use of alarms. In T2D, unlike T1D, not all the functions of the CGM need to be activated, and the system seems more appropriate for the retrospective evaluation by using the electronic diary and events logging rather than real time assessment. The system should be adapted and personalised according to each person with T2D, ongoing treatment, and decisions to avoid the risk of inadequate management and to optimise the benefit of the technology.

Although CGM is known for its metabolic benefits in managing blood glucose levels, its psychological impact should not be overlooked. By providing real‐time glucose data, CGM significantly reduces the uncertainty and anxiety associated with blood sugar fluctuations, particularly in individuals who experience fear of hypoglycemia [90]. This continuous stream of data empower patients to take immediate action, enhancing their confidence in managing their condition [90]. Furthermore, CGM reduces the need for frequent fingerstick testing, which can alleviate discomfort and the emotional burden of routine glucose monitoring [90]. This reduction in testing frequency, combined with the ability to monitor glucose trends remotely, not only improves treatment adherence but also enhances the overall quality of life by offering patients and their healthcare providers greater flexibility and support in managing diabetes [90]. These psychological benefits can also contribute to reduced stress levels and improved long‐term outcomes, as patients are more likely to engage with their treatment plan and maintain better glucose control [90]. While CGM has been shown to improve the QoL and satisfaction for patients with both T1D and T2D, as evidenced by various QoL questionnaires [39], these studies did not specifically address anxiety related to CGM use. Alarm‐related anxiety, a well‐documented drawback of CGM, was previously discussed in relation to adherence strategies. Anxiety may also arise from the pressure to achieve optimal TIR. In T1D, research suggests that anxiety is often associated with hyperglycaemia, where the relationship may be bidirectional: anxiety can both cause hyperglycaemia and be a consequence of it. A recent Italian study highlights the connection between CGM metrics and psycholinguistic measures, suggesting that poor TIR may be linked to higher levels of anxiety [91]. To mitigate anxiety, strategies such as alarm customisation, psychological support, and realistic goal‐setting can be employed to help patients manage stress and improve overall CGM adherence and outcomes.

## 7 Cost‐Effectiveness Analysis: CGM and Basal Insulin Treated Patients With T2D

A recent study assessed the cost utility from an Italian healthcare system perspective of isCGM, compared with SMBG, in people with T2D using basal insulin by means of the DEDUCE (DEtermination of Diabetes Utilities, Costs, and Effects) microsimulation model [92]. The model was run for 10,000 patients over a lifetime horizon of 50 years, based on Italian population data, randomized controlled trials, and a real‐world database. The base‐case incremental cost‐effectiveness ratio (ICER) for isCGM versus SMBG was €6641/QALY. Total costs were €4602 higher with isCGM than with SMBG (€62,085 vs. €57,483). isCGM was associated with an additional 0.69 QALYs versus SMBG (13.71 vs. 13.02). Scenario analysis ICERs ranged from €2433/QALY to €17,227/QALY. The highest ICERs were seen when the model time horizon was reduced to 5 years and when lower reductions in HbA1c were applied. In the probabilistic analysis, isCGM was 86% likely to be cost‐effective at a willingness‐to‐pay threshold of €10,000/QALY, and 100% likely at thresholds > €15,000/QALY. This economic evaluation demonstrated that basal insulin glucose monitoring with isCGM is a cost‐effective option for people with T2D compared with current care standards in Italy. Probabilistic sensitivity analysis found a 100% likelihood of isCGM being cost effective at willingness‐to‐pay thresholds above €15,000. In addition, the results were generally consistent across all scenarios tested. The largest increase in ICER was found when HbA1c reductions were taken from an RCT evaluating the use of isCGM by patients with T2D using non‐insulin treatment [61]—although this scenario is conservative, the resultant ICER is still likely to be considered cost effective from an Italian healthcare system perspective. To conclude, from an Italian healthcare system perspective, isCGM can be considered to be cost effective compared with SMBG for people with T2D using basal insulin therapy [93].

A retrospective analysis of the MarketScan Databases was conducted to compare healthcare resource utilization (HCRU) and costs between SMBG and CGM users in adults with non‐intensively managed T2D using propensity score matching. A total of 3498 patients were included in each matched cohort. Analysis of claims data suggests that SMBG users appear to have lower all‐cause costs compared with CGM users. Indeed, the per‐patient per‐year (PPPY) all‐cause cost was $20,542 in CGM users, versus $19,349 in SMBG users (*p* < 0.001). Findings suggest that the use of SMBG is less costly than CGM in this patient population mainly because of lower pharmacy costs, including costs for glucose‐lowering medications. Furthermore, no significant differences in the number of emergency department visits or hospitalizations were observed, but CGM users had more all‐cause outpatient visits and office visits with an endocrinologist [94].

## 8 Conclusions

T2D presents a global health challenge with significant implications for patient management and healthcare systems. Effective treatment adherence is crucial for achieving glycaemic control and minimising complications. CGM technology has emerged as a pivotal tool in enhancing patient empowerment and management of T2D.

Our analysis confirms that continuous CGM provides substantial benefits for individuals with T2D, particularly those on MDI or basal insulin therapy. Continuous CGM use has been consistently associated with improved glycaemic control, as evidenced by reductions in HbA1c levels and enhanced TIR. This technology supports patients in making informed decisions about their glucose management, thus potentially reducing the risk of hypo‐ and hyperglycaemic episodes.

While the integration of CGM technology into routine care is evolving, its demonstrated advantages justify its broader implementation. Economic evaluations indicate that CGM is a cost‐effective option compared to standard SMBG, especially when considering long‐term health outcomes and QoL improvements.

On the other hand, this expert opinion presents some new findings and highlights the potential need for intermittent CGM use in selected cases of patients taking oral antidiabetic drugs (e.g., for therapy titration or intensification, after diagnosis, for diet planning, before and after surgery, in case of gastroparesis, during pregnancy).

In summary, the continuous use of CGM offers a significant advantage in managing T2D by facilitating better glycaemic control and patient engagement. The evidence supports its role as an essential component in diabetes care, contributing to more effective management strategies and improved patient outcomes.

## Author Contributions

C.I. was responsible for drafting the manuscript. The other authors provided supervision and critical revisions. All authors have read and approved the final version of the manuscript.

## Conflicts of Interest

CI has provided advisory board services for Novo Nordisk, Roche Diabetes Care Italy, Abbott, Menarini, Ascensia, and Senseonics and has received speaker fees from Novo Nordisk, Roche Diabetes Care Italy, Abbott, Ascensia, Lilly, and Boehringer Ingelheim Pharmaceuticals; FB received honoraria and speaker fees from Abbott, Medtronic, Theras, Movi, Lilly; SDP has served as president of EASD/European Foundation for the Study of Diabetes (EFSD) (2020–2022) and is the president of the Menarini Foundation; has received research grants to the institution from AstraZeneca and Boehringer Ingelheim; has served as advisor for Abbott, Amarin Corporation, Amplitude, Applied Therapeutics, AstraZeneca, Biomea Fusion, Eli Lilly & Co., EvaPharma, Menarini International, Novo Nordisk, Sanofi, and Sun Pharmaceuticals; and has received fees for speaking from AstraZeneca, Boehringer Ingelheim, Eli Lilly & Co., Laboratori Guidotti, Menarini International, Merck Sharpe & Dohme, and Novo Nordisk; PDB received honoraria and speaker fees from Abbott, Medtronic, Theras, Eli Lilly, Novo Nordisk, Guidotti, Boehringer, Astra Zeneca, Ascensia Bayer, Allergan, Insulet; FG: Eli Lilly, Roche Diabetes Care (grants); Eli Lilly, Novo Nordisk (consulting fees); AstraZeneca, Boehringer‐Ingelheim, Eli Lilly, Lifescan, Merck Sharp & Dohme, Medtronic, Novo Nordisk, Roche Diabetes Care, Sanofi Aventis; Eli Lilly, Sanofi Aventis (support for attending meetings/travel); AstraZeneca, Boehringer‐Ingelheim, Eli Lilly, Lifescan, Merck Sharp & Dohme, Medimmune, Medtronic, Novo Nordisk, Roche Diabetes Care, Sanofi Aventis (participation on Advisory Boards); EASD/EFSD, Società Italiana di Endocrinologia (SIE), Fo.Ri.SIE (unpaid leadership); AstraZeneca, Eli Lilly, Novo Nordisk, Sanofi Aventis (support for medical writing and statistical analysis); AA, RB, RC, SDP, PF, CBG have no competing interests to declare. The sponsor played no role in the design, execution, interpretation or writing of this manuscript.

## Peer Review

The peer review history for this article is available at https://www.webofscience.com/api/gateway/wos/peer-review/10.1002/dmrr.70059.

## Data Availability

Data sharing is not applicable to this article as no new data were created or analysed in this study.
